# Hypoxia-induced endoplasmic reticulum stress characterizes a necrotic phenotype of pancreatic cancer

**DOI:** 10.18632/oncotarget.5168

**Published:** 2015-10-05

**Authors:** Bo Kong, Tao Cheng, Weiwei Wu, Ivonne Regel, Susanne Raulefs, Helmut Friess, Mert Erkan, Irene Esposito, Jörg Kleeff, Christoph W. Michalski

**Affiliations:** ^1^ Department of Surgery, Technische Universität München (TUM), Munich, Germany; ^2^ Institute of Pathology, Medical University Innsbruck, Innsbruck, Austria; ^3^ Department of Surgery, Koc University School of Medicine, Istanbul, Turkey; ^4^ Department of Surgery, University of Heidelberg, Heidelberg, Germany

**Keywords:** ER stress, VEGFA, hypoxia, necrosis, pancreatic cancer

## Abstract

Stromal fibrosis and tissue necrosis are major histological sequelae of hypoxia. The hypoxia-to-fibrosis sequence is well-documented in pancreatic ductal adenocarcinoma (PDAC). However, hypoxic and necrotic PDAC phenotypes are insufficiently characterized. Recently, reduction of tuberous sclerosis expression in mice together with oncogenic Kras demonstrated a rapidly metastasizing phenotype with histologically eccentric necrosis, transitional hypoxia and devascularisation. We established cell lines from these tumors and transplanted them orthotopically into wild-type mice to test their abilities to recapitulate the histological features of the primary lesions. Notably, the necrotic phenotype was reproduced by only a subset of cell lines while others gave rise to dedifferentiated tumors with significantly reduced necrosis. *In vitro* analysis of the necrotic tumor-inducing cell lines revealed that these cells released a significant amount of vascular endothelial growth factor A (VEGFA). However, its release was not further increased under hypoxic conditions. Defective hypoxia-induced VEGFA secretion was not due to impaired VEGFA transcription or hypoxia-inducible factor 1-alpha activation, but rather a result of hypoxia-induced endoplasmic reticulum (ER) stress. We thus identified hypoxia-induced ER stress as an important pathway in PDACs with tissue necrosis and rapid metastasis.

## INTRODUCTION

Pancreatic ductal adenocarcinoma (PDAC) is notoriously aggressive and hypoxic [1, 2]. As the extent of hypoxia has been reported to be associated with the prognosis of PDAC patients [3–5], it constitutes an important aspect of its lethal biology. Indeed, hypoxia fosters a variety of malignant features of PDAC by 1) promoting the metastatic spread [6]; 2) facilitating the acquisition of stem cell-like properties [7, 8], and 3) limiting the delivery of conventional chemotherapeutic agents [9]. Thus, targeting tumor hypoxia by using hypoxia-activated substances (or bioreductive prodrugs, e.g. TH-302) has emerged as a promising strategy to improve the outcome of PDAC patients [10].

Stromal fibrosis and tissue necrosis are major histological sequelae of hypoxia in PDAC [11–13], probably reflecting the biological consequences of dissociated tumor expansion and insufficient neo-angiogenesis. We have previously provided *in vitro* evidence suggesting that an interaction between cancer cells and pancreatic stellate cells (PSCs) initiates and perpetuates a fibrotic, hypoxic and tumor-supportive microenvironment [11, 14]. This notion has recently been challenged by experimental data generated *in vivo* [15, 16]. Despite the fact that different transgenic mouse models and different approaches for depleting fibrosis were used, two independent studies reached the same conclusion that the fibrotic component of stroma restrained tumor growth rather than supporting it. However, the role of fibrosis in angiogenesis and hypoxia is largely inconsistent: disruption of fibrosis promoted angiogenesis in one study [16] while it inhibited angiogenesis and aggravated tissue hypoxia in another one [15]. Therefore, fibrosis, hypoxia and impaired angiogenesis play complex and not always concerted roles in pancreatic carcinogenesis.

The less well characterized hypoxic/necrotic phenotypes of PDAC add further complexity. Previous descriptive studies in human PDAC have demonstrated that micro- and macro-necrosis can be observed in the majority of cases and that the presence of tumor necrosis is associated with unfavourable prognosis, implying a potential tumor-promoting role associated with the hypoxic/necrotic phenotype [12, 13]. Indeed, we previously characterized a murine model of highly metastatic PDAC driven by oncogenic Kras/Mek-mTOR (mechanistic target of rapamycin) signalling in which the observed massive tumor necrosis was associated with metastatic spread [17]. In the current study, we aimed at uncovering potential molecular mechanisms underlying the hypoxic/necrotic phenotype.

## RESULTS

### Identification of a hypoxic/necrotic tumor phenotype

**Figure 1:**
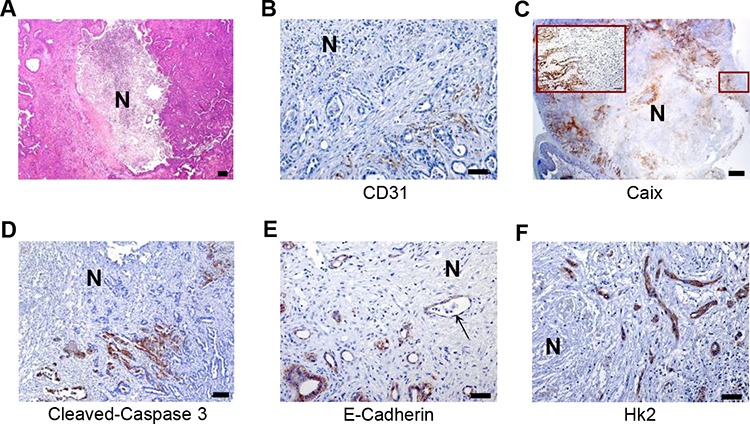
Identification of a hypoxic/necrotic tumor phenotype **A.** Representative H&E-stained sections of necrotic PDACs; scale bar: 100 μm, **B–D.** IHC studies of CD31, Caix and cleaved-caspase 3 as surrogate markers for vessel localization (scale bar: 50 μm), hypoxic regions (scale bar: 500 μm) and apoptotic cells (scale bar: 100 μm) in metastatic PDACs. **E–F.** IHC studies for E-Cadherin and Hk2 show loss of membrane staining of E-Cadherin (arrow) and increased expression of Hk2 in cancer cells in the vicinity of tissue necrosis (also hypoxic regions) in metastatic PDACs, scale bar: 50 μm, N: necrosis.

Histological examination of PDAC tissue resulting from oncogenic Kras/Mek-mTOR signalling in a mouse model revealed large and eccentric necrosis surrounded by avascular areas [17] (Figure 1A). To further characterize tumor histology, we labelled vessels using CD31 antibodies. This analysis revealed that the necrotic region was surrounded by devascularized tumor areas (Figure 1B) in which cancer cells were specifically carbonic anhydrase IX- (Caix; a hypoxia marker) and cleaved-Caspase 3-positive (Figure 1C, 1D). In addition, other hypoxia-induced molecular changes were also observed: membrane expression of E-Cadherin was lost in cancer cells close to devascularized areas. Furthermore, acquisition of a fibroblastic characteristic (resembling features of epithelial-to-mesenchymal transition (EMT)) and increased expression of Hk2, a surrogate marker of Hif1α activation, was seen (Figure 1E, 1F). Collectively, these results demonstrated a tumor histology in this mouse model closely resembling the human hypoxic/necrotic PDAC phenotype [12, 13].

### *In vivo* screening of PDAC cells carrying the hypoxic/necrotic phenotype

**Figure 2:**
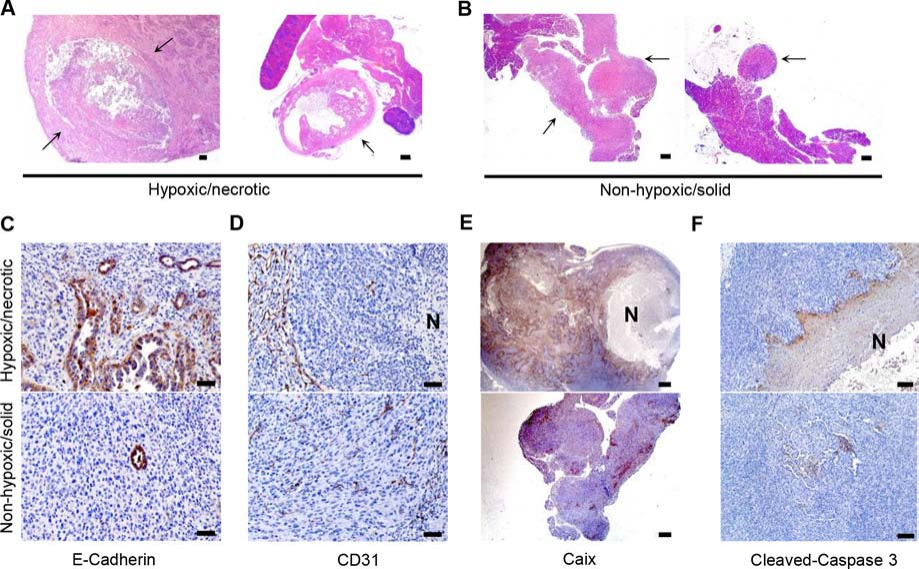
*In vivo* screening of PDAC cells with an *in vivo* preserved hypoxic/necrotic phenotype **A–B.** H&E–stained sections show centrally-necrotic tumors formed after orthotopic implantation of the hypoxic/necrotic cells and more solid forms of tumors formed by dedifferentiated cells, scale bar: 500 μm; arrow: tumor tissues; **C.** IHC with anti-E-Cadherin demonstrates a high proportion of anaplastic components in tumors developing from the hypoxic/necrotic cells, but not in tumors developing from the dedifferentiated cells, scale bar: 50 μm; **D–F.** IHC of anti-CD31 (scale bar: 50 μm), anti-Caix (scale bar: 500 μm) and anti-cleaved-caspase 3 (scale bar: 100 μm) show devascularized tumor regions, hypoxic zones and apoptosis in the necrotic tumors forming after transplantation of the hypoxic/necrotic cells, but not in the anaplastic tumors forming after transplantation of the dedifferentiated cells; N: necrosis.

To test whether this particular phenotype was preserved in isolated PDAC cell lines, we transplanted a number of these cell lines (*n* = 6, established from above described mouse models) orthotopically into wild type (WT) mice to test their ability to recapitulate the histology of the primary tumor. Five cell lines (399, 403, 445Li, 907 and 908Li) formed tumors at a 100% (35/35) penetrance while only 3 out of 7 (43%) of the 897 cell-transplanted mice developed tumors. Clear differences in tumor histology were observed: the 399, 403 and 445Li cells gave rise to PDAC characterized by large areas of central necrosis (Figure 2A); the 907, 908Li and 897 cells developed into solid tumors (Figure 2B). The solid tumors contained a higher proportion of dedifferentiated/sarcomatoid-like components than the necrotic ones (Figure 2C; as exemplified by E-Cadherin Immunohistochemistry (IHC)). The necrotic tumors recapitulated the core histological features of the hypoxic/necrotic phenotype including devascularized tumor areas, hypoxia and apoptosis (Figure 2D, 2E and 2F, upper panel). No such features were observed in the histology of solid tumors (Figure 2D, 2E and 2F, lower panel). Thus, the cell lines giving rise to the necrotic tumors with features of hypoxia were labelled “hypoxic/necrotic” whereas the cell lines forming solid dedifferentiated tumors without pronounced features of hypoxia and significantly less necrosis were labelled “non-hypoxic/solid”. In addition, *Kras* sequencing of these cell lines revealed that the hypoxic/necrotic cells were heterozygous for Kras^G12D^ while the non-hypoxic/solid contained only one mutated allele, showing loss of heterozygosity (LOH) at the Kras locus. PCR analysis confirmed loss of the WT Kras allele in these cells (Supplementary Figure 1A), comparable to previous reports [18]. Western-blot analyses and Ras pull-down assays revealed that neither Kras expression nor the steady-state/serum-induced Kras activity (GTP-Kras) were affected by Kras^G12D^-LOH (Supplementary Figure 1B).

### Compromised hypoxia-driven VEGFA secretion in PDAC cells with a hypoxic/necrotic phenotype

**Figure 3:**
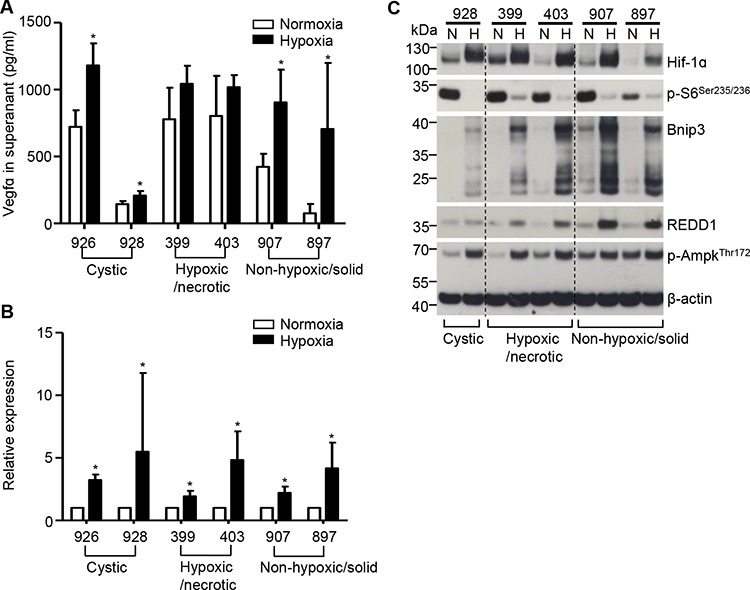
Compromised hypoxia-driven VEGFA secretion in PDAC cells with the hypoxic/necrotic phenotype **A.** ELISA assays show the release of VEGFA into the supernatants of hypoxic/necrotic cells (399 and 403 cells), dedifferentiated cells (907 and 897 cells) and “cystic” cells (926 and 928 cells) after exposure to hypoxia for 24 hours. Values shown are obtained from at least three independent experiments; *p < 0.05; **B.** VEGFA mRNA levels (measured by QRT-PCR) after induction of hypoxia in all tested cell lines. Data are presented as relative expression (normalized to the median of the respective expression level under normoxia); an unpaired *t*-test was performed; values shown are obtained from at least three independent experiments; *p < 0.05; C, Western-blot analysis shows expression of Hif1alpha, p-S6^Ser235/236^, Bnip3, Redd1 and p-Ampk^Thr172^ in hypoxic/necrotic cells (399 and 403 cells), dedifferentiated cells (907 and 897 cells) and “cystic” cells (928 cells) after exposure to hypoxia for 24 hours; N: normoxia, H: hypoxia; one representative blot out of three independent experiments is shown.

As hypoxia-driven angiogenesis relies on the secretion of a plethora of hypoxia-induced angiogenic factors such as most importantly VEGFA, we measured the release of VEGFA following a 24 hour-exposure to hypoxia in hypoxic/necrotic cells (399 and 403 cells). The non-hypoxic/solid cells (907 and 897 cells) and so-called cystic cell lines established from previously described cystic tumors (926 and 928 cells [17]: “cystic”) were used as internal and external controls, respectively. Notably, the hypoxic/necrotic cells secreted high levels of VEGFA but did not increase VEGFA secretion after exposure to hypoxia; however, both the non-hypoxic/solid and cystic cell lines had no such “defect” (Figure 3A). Delineation of the underlying mechanisms demonstrated that hypoxia-induced VEGFA transcription and Hif1alpha activation were significantly increased in all cell lines including the hypoxic/necrotic cells (Figure 3B, 3C). In addition, the hypoxia-induced responses such as dephosphorylation of S6 (p-S6^Ser235/236^), phosphorylation of Ampk (p-Ampk^Thr172^) and expression of Bnip3 (BCL2/adenovirus E1B interacting protein 3) or of REDD1 were not altered in hypoxic/necrotic cells (Figure 3C). Taken together, these data demonstrate that hypoxia-driven VEGFA secretion is compromised in PDAC cells with the hypoxic/necrotic phenotype; additionally, such defects are not due to impaired VEGFA transcription or Hif1α activation.

### Sensitivity of the hypoxic/necrotic cells to hypoxia-induced ER stress

**Figure 4:**
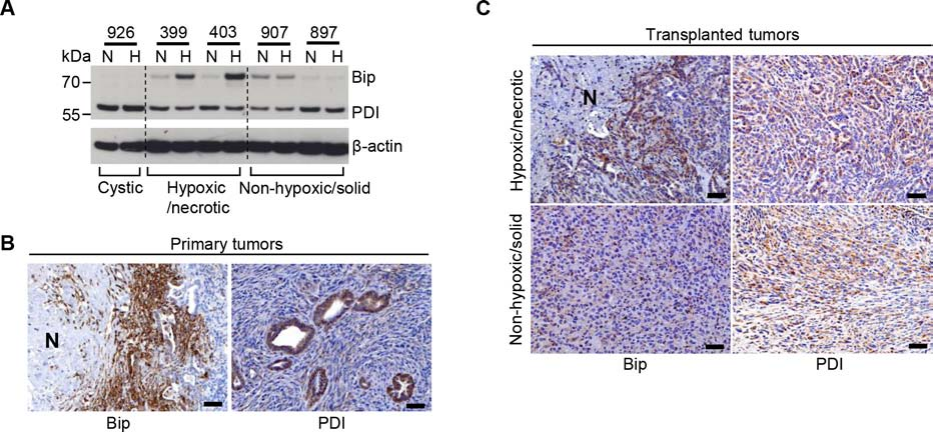
Sensitivity of the hypoxic/necrotic cells to hypoxia-induced ER stress **A.** Western-blot analysis demonstrates induced expression of Bip and PDI in hypoxic/necrotic cells (399, 403), in dedifferentiated cells (907, 897) and in cystic cells (926) after exposure to hypoxia for 24 hours; N: normoxia, H: hypoxia; one representative blot out of three independent experiments is shown, **B.** Representative IHC pictures show expression of Bip and PDI in metastatic PDACs, scale bar: 50 μm, **C.** Representative IHC pictures show expression of Bip and PDI in the transplanted tumor tissues induced by transplantation of hypoxic/necrotic or dedifferentiated cells, scale bar: 50 μm.

Because secretion and folding of VEGFA rely on the formation of a disulfide bond (an oxidative reaction) in the endoplasmic reticulum (ER) [19, 20], it is likely that the VEGFA secretion in ER is highly vulnerable to oxygen deprivation. Here, Bip (heat shock protein 5), a marker of ER stress, was indeed induced by hypoxia in hypoxic/necrotic cells. The non-hypoxic/solid and cystic cell lines were more resistant to hypoxia-induced ER stress (Figure 4A). Expression of the protein disulfide isomerase (PDI) responsible for disulfide formation remained unchanged under hypoxic conditions (internal control, Figure 4A). In line with these findings, Bip expressing cells were specifically detected in the hypoxic, devascularized regions of primary hypoxic/necrotic PDACs (Figure 4B) and of transplanted hypoxic/necrotic tumors (Figure 4C). No such patterns were observed in the transplanted dedifferentiated tumors (Figure 4B, 4C). Besides, no differences in PDI expression were found comparing these tumor entities (Figure 4B, 4C). Taken together, the hypoxic/necrotic cells were highly sensitive to hypoxia-induced ER stress.

## DISCUSSION

In general, hypoxia constitutes an important source of ER stress and activates the unfolded protein response (UPR) system to mediate a set of adaptive changes within solid tumors [21, 22]. However, why protein secretion is impaired and how ER stress is induced by hypoxia is not clear. One of the potential reasons is that oxygen provides the ultimate oxidative potential for disulfide formation, which is essential for the folding of secretory proteins. VEGFA is a typical example: the folding and proper function of VEGFA requires both intramolecular and intersubunit disulfide bonds. Therefore, secretion of VEGFA relies on the maintenance of the oxidative potential of the ER [20]. Upon oxygen deprivation, unfolded VEGFA (or other secreted proteins) accumulate in the ER and cause ER stress and UPR. This is especially important for cancer cell lines with high levels of VEGFA secretion. Here, the oxygen supply needs to be constantly maintained to support oxidative folding of high-throughput VEGFA secretion. Subtle changes in oxygen supply may disturb proper folding of VEGFA and cause a severe “traffic jam” along the secretory pathway. This partially explains why cancer cells with high levels of VEGFA secretion are susceptible to hypoxia-induced ER stress.

Interestingly, the secretion of VEGFA is not the only determinant factor: one cystic cell line sustained by the oncogenic PI3K/Akt-mTOR signal secretes (926 cells) a significant amount of VEGFA [17], however, no hypoxic/necrotic phenotype was observed. Importantly, this cell line was resistant to hypoxia-induced ER stress. These data demonstrate that it is not the level of VEGFA secretion that correlates with the hypoxic/necrotic phenotype. Instead, the hypoxia-induced ER stress correlates with the hypoxic/necrotic phenotype. Whether this correlation constitutes a causal link requires further investigations. In particular, the functional relevance of VEGFA secretion in the development of hypoxic/necrotic phenotype seems to be largely context-dependent and complex (see below).

In line with previous reports [23], the hypoxic/necrotic cells sustained by the oncogenic Kras/Mek-mTOR signal produced relatively high levels of VEGFA. It is conceivable that an angiogenic switch can easily be achieved by hypoxic/necrotic cells at the incipient stage of carcinogenesis. However, as the tumor volume increases, hypoxia invariably occurs, which readily triggers ER stress in these cells due to its intrinsic sensitivity. The underlying reasons for this intrinsic sensitivity and the biological relevance of such hypoxia-induced ER stress requires further elucidation. In particular, it is unclear whether it contributes to the aggravation of local hypoxia by compromising VEGFA secretion, whether it is responsible for cell death, or whether it promotes the metastatic spread of PDAC. Interestingly, we observed that a subset of cancer cells appeared to lose the hypoxic/necrotic phenotype with a concomitant acquisition of LOH status at Kras locus due to unknown reasons. To clarify these questions warrants further investigation. Nevertheless, these data suggest that the hypoxia-induced ER stress constitutes a potential target for ameliorating the hypoxic/necrotic phenotype. In this regard, we recently identified a subtype of human PDAC with high ER stress levels which is labelled by a common variant of MIA2 gene (melanoma inhibitory activity 2, MIA2^I141M^, [24]). Certainly, it would be worthwhile to analyse whether the hypoxic/necrotic phenotype is more dominant in this PDAC subtype.

## MATERIALS AND METHODS

### Mouse breeding and collection of mouse tissues

Mouse breeding and tissues collection were described previously [17]. Briefly, the metastatic/necrotic PDACs developed in triple transgenic *p48^Cre/+^; LSL-Kras^G12D/+^; Tsc1^flox/+^* compound mice while the cystic tumors were derived from triple transgenic *p48^Cre/+^; Pten^flox/flox^; Tsc1^flox/+^* compound mice. Wild-type (WT; C57BL/6J) mice were obtained from Charles River Laboratory (Sulzfeld, Germany). Mouse breeding was performed and husbandry was maintained at the specific pathogen free (SPF) mouse facility at the Technical University of Munich. The compound transgenic mice were maintained on a mixed background. All mouse experiments and procedures were approved by Bavarian Government (No. 55.2–1-54–2532-42–13). All procedures were in accordance with the Office of Laboratory Animal Welfare and the German Federal Animal Protection Laws.

### Cell transplantation experiments

Orthotopic transplantations of mouse cells were carried out as described [25]. Briefly, mice were anesthetized, and a left-lateral incision of the abdomen was made to visualize the tail region of the pancreas. 10^6^ cells suspended in 50 μl of PBS were carefully injected into the pancreatic tail. The abdominal wall and skin were closed using running sutures. All mice were sacrificed for histological evaluation after 4 weeks.

### Hypoxia assays

Pancreatic cancer cell lines were incubated in a modular chamber saturated with a hypoxic air mixture (89.25% N_2_ + 10% CO_2_ + 0.75% O2) for 24 h at 37°C. For measurements of VEGFA, serum-free medium was used. The remaining experiments were performed in medium supplemented with 10% FCS. All experiments were repeated three times.

### Further materials and methods

A detailed materials and methods section is provided as a supplement to this manuscript.

### Statistical analysis

Either GraphPad Prism 5 Software (GraphPad, San Diego, CA, USA) was used for the statistical analysis. Unless otherwise stated, an unpaired *t*-test was used for group-wise comparisons. Statistical significance was set at *p* < 0.05.

## SUPPLEMENTARY DATA FIGURE



## Abbreviations

PDAC: pancreatic ductal adenocarcinoma
ER: endoplasmic reticulum
VEGFA: vascular endothelial growth factor a
Tsc: Tuberous sclerosis complex
mTOR: mammalian target of rapamycin
PI3K: phosphatidylinositol-4, 5-bisphosphate 3-kinase
Hif1α: hypoxia-inducible factor 1 alpha
