# Supplementary material for: Hypoxia-induced endoplasmic reticulum stress characterizes a necrotic phenotype of pancreatic cancer
Source: Oncotarget. 2015 Oct 5;6(31):32154–60. doi: 10.18632/oncotarget.5168 (PMC4741665; doi:10.18632/oncotarget.5168)
Supplement: Supplementary file 1 [file oncotarget-06-32154-s001.pdf]

SUPPLEMENTARY DATA

MATERIALS AND METHODS

Mouse cell lines

926, 928 and 952 cells were isolated from transgenic *p48Cre/+; Ptenflox/flox; Tsc1flox/+*mice, respectively. 399, 403, 907, 897, 445Li, 445Pa, 638Li, 908Pa, 908Li and 908Lu cells were isolated from transgenic *p48Cre/+; LSL-KrasG12D/+; Tsc1flox/+* mice.

Primer sequences

Sequences of primers used for QRT-PCR analysis of the mouse genes

Gene Name	Sense (5′ → 3′)	Antisense (5′ → 3′)
Vegfa	ACT CGG ATG CCG ACA CGG GA	CCT GGC CTT GCT TGC TCC CC
B2m	CGG CCT GTA TGC TAT CCA GAA AAC C	TGT GAG GCG GGT GGA ACT GTG

List of antibodies

Primary antibodies

Antibody name	Catalog number	Application* (Reactivity**)	Producer
Rabbit Anti-PDI mAb#	3501	WB; IHC (M)	Cell Signaling Technology (NEB, Frankfurt/ Main,Germany)
Rabbit Anti-BiP mAb#	3177	WB; IHC (M)	Cell Signaling Technology
Rabbit Anti-p-S6$^{Ser235/236}$ Ab#	2211	WB (M)	Cell Signaling Technology
Rabbit Anti-Cleaved Casp3 mAb#	9664	IHC (M)	Cell Signaling Technology
Rabbit Anti-Bnip3 Ab#	3769	WB (M)	Cell Signaling Technology
Rabbit Anti-E-cadherin mAb#	3195	WB; IHC (M)	Cell Signaling Technology
Rabbit Anti-Hk2 mAb#	2867	WB; IHC (M)	Cell Signaling Technology
Rabbit Anti-p-AMPKα^{Thr172} mAb#	2535	WB (M)	Cell Signaling Technology
Mouse Anti-Hif1α Ab#	NB100–105	WB (M)	Novus Biologicals (Cambridge, UK)
Rabbit Anti-CAIX Ab#	ab15086	IHC (M)	Abcam (Cambridge, UK)
Rat Anti-CD31 Ab#	DIA-310	IHC (M)	Dianova (Hamburg, Germany)
Rabbit Anti-GAPDH Ab#	sc-25778	WB (M)	Santa Cruz biotechnology (Heidelberg, Germany)
Mouse Anti-β actin Ab#	sc-69879	WB (M)	Santa Cruz biotechnology
Rabbit Anti-REDD1 pAb#	10638–1-AP	WB (M)	Proteintech (Manchester, UK)

Secondary antibodies

Antibody name	Catalog number	Application*	Producer
Goat HRP-Labelled Polymer Anti-Mouse Ab#	K4001	IHC	Dako Deutschland GmbH
Goat HRP-Labelled Polymer Anti-Rabbit Ab#	K4003	IHC	Dako Deutschland GmbH
Sheep HRP-labelled Anti-Mouse IgG Ab#	NA931	WB	GE Healthcare (Little Chalfont, UK)
Donkey HRP-labelled Anti-Rabbit IgG Ab#	NA934	WB	GE Healthcare

*Application key: WB = western-blot; IHC = Immunohistochemistry;
**Reactivity key: M = mouse;
#Ab: antibody

Immunohistochemistry analysis

Immunohistochemistry was performed using the Dako Envision System (Dako Cytomation GmbH, Hamburg, Germany). Consecutive paraffin-embedded tissue sections (3–5 mm thick) were deparaffinized and rehydrated using routine methods. Antigen retrieval was performed by pretreatment of the slides in citrate buffer (pH 6.0; 10 mM Citric Acid, 0.05% Tween 20) in a microwave oven for 10 minutes. Endogenous peroxidase activity was quenched by incubation in deionized water containing 3% hydrogen peroxide at room temperature for 10 minutes. After blocking of nonspecific reactivity with TBS (pH 7.4; 0.1M Tris Base, 1.4M NaCl) containing 3% BSA or goat serum, sections were incubated with the respective antibody at 4°C overnight followed by incubation with horseradish peroxidase-linked goat anti-rabbit or mouse antibodies, followed by a color-reaction with diaminobenzidine and counterstaining with Mayer's hematoxylin.

Immunoblot analysis

Protein extraction from cells

Cells were washed twice with ice-cold PBS (pH 7.4; 0.01M PBS). After addition of ice-cold modified RIPA buffer/or cell lysis buffer containing 20 mM Tris-HCl (pH 7.5), 150 mM NaCl, 1 mM Na_2EDTA, 1 mM EGTA, 1% NP-40, 1% sodium deoxycholate, 2.5 mM sodium pyrophosphate, 1 mM β-glycerolphosphate, 1 mM $NaVO_4$, 1 μg/ml leupeptin and 1 mM PMSF, cells were immediately homogenized by passing them through a G27 syringe needle 10 times. The crude homogenate was then centrifuged at 14,000 g in a pre-cooled centrifuge for 15 minutes. The supernatant was immediately transferred to fresh tubes and was aliquoted. The concentration of the extracted protein was determined using the BCA Protein Assay kit (Pierce, Thermo Scientific, USA) following the manufacturer's instructions. The sample aliquots were stored at −20°C or were used for Western blotting analysis immediately.

Western blotting

20 to 80 μg of the total protein were loaded on 4 ~ 12% polyacrylamide gels and were transferred to PVDF membranes. Membranes were blocked in 20 ml Tween-20 (0.05%)-TBS, 3% or 5% skim milk and 0.05% Tween-20 for 1 hour and were incubated with the respective primary antibody overnight at 4°C. Membranes were washed 3 times with 0.05% Tween-20-TBS and were incubated with a horseradish peroxidase (HRP)-conjugated secondary antibody (1:3000) for 1 h at room temperature. Signals were detected using the enhanced chemiluminescence system (ECL, Amersham Life Science Ltd., Bucks, UK). Films were scanned with a CanoScan 9900F scanner (Canon, Tokyo, Japan).

Cell culture and treatment

Human or mouse cell lines were cultured in 10 cm dishes either in DMEM or RPMI-1640 cell culture medium supplemented with 10% fetal bovine serum (FBS), 100 u/ml penicillin and 100 μg/ml streptomycin at 37°, 5% CO2.

ELISA

For the measurement of Vegfa secretion of mouse cells, cells were cultured with 1 ml of serum-free medium in 6-well plates for 24 hours under normoxic or hypoxic

conditions. The Vegfa concentration was determined using a commercially available kit ("Mouse VEGF Quantikine ELISA Kit", MMV00, R&D Systems, WIESBADEN, Germany).

Primary cell isolation

Freshly dissected sterile tumor tissues were washed twice with ice-cold PBS, were cut into small pieces (approximately 1 mm) and dispensed into 5 ml of complete medium containing collagenase (1.2 mg/ml). The resulting solution (mixed with tissue blocks) was incubated at 37°C for 0.5 hours. After centrifugation at 300 rpm for 5 minutes, small tissue blocks were washed twice with collagenase-free medium, followed by incubation at 37°C with medium containing collagenase for additional 0.5 hours. After passing the undigested tissue blocks through a 100 µm nylon mesh, cell suspensions were obtained. These cell suspensions were washed two times with complete medium and were seeded into a 10 cm^2 dish.

MRNA and cDNA preparation

All reagents for RNA extraction and cDNA transcription were from Qiagen (Hilden, Germany) and were used according to the manufacturer's instructions.

Quantitative Real-Time Polymerase Chain Reaction

Quantitative real time PCR (QRT-PCR) was carried out using the LightCycler™480 system with the SYBR Green 1 Master kit (Roche diagnostics). Expression of the target gene was normalized to the human housekeeping genes ACTB (β-actin) and HPRT1 (Hypoxanthine phosphoribosyltransferase 1) or the mouse housekeeping gene B2m (β-2 microglobulin) using the LightCycler™480 software release 1.5, version 1.05.0.39 (Roche diagnostics).

Active Ras pull-down assay

Cells were cultured in serum-free medium for 24 hours followed by treatment with 20% FBS or serum-free medium for 5 minutes. After treatment, active Kras was detected using the Active Ras Pull-Down and Detection Kit (THERMO SCIENTIFIC, Waltham, USA) according to the manufacturer's instruction. 500 µg of total protein were used for the pull-down reaction.

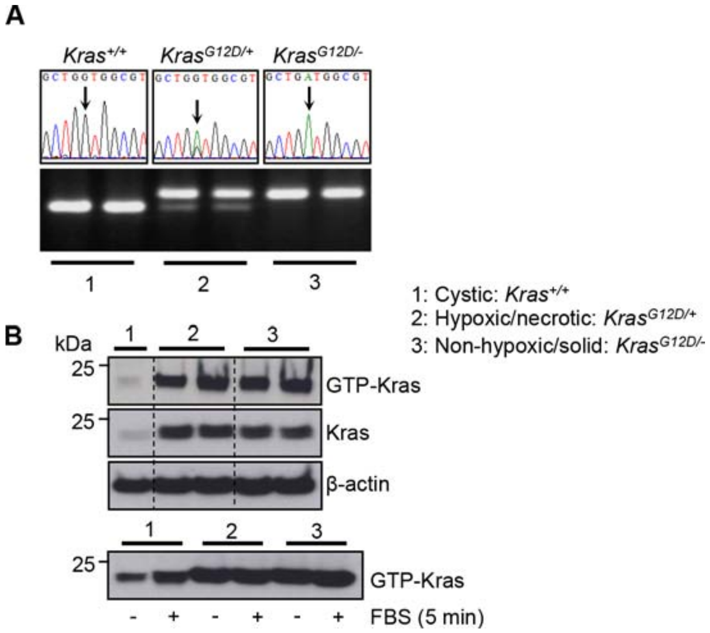

Supplementary Figure S1: A. Sequencing and PCR demonstrate a Kras^{G12D}-LOH in the hypoxic/necrotic cells, but not in the non-hypoxic/solid cells; two cystic cell lines contain only WT Kras alleles; B. Ras pull-down assays show that the cancer cells with the Kras^{G12D}-LOH have a comparable steady-state (upper panel) and serum-stimulated Kras activity (low panel) as the Kras^{G12D} cancer cells without the LOH. The cystic cells with the WT Kras alleles were used as a negative control for these assays; one out of three independent experiments was shown.
